# Functional Interactions of Tau Phosphorylation Sites That Mediate Toxicity and Deficient Learning in *Drosophila melanogaster*

**DOI:** 10.3389/fnmol.2020.569520

**Published:** 2020-10-21

**Authors:** Iason Keramidis, Ergina Vourkou, Katerina Papanikolopoulou, Efthimios M. C. Skoulakis

**Affiliations:** ^1^Biomedical Sciences Research Centre “Alexander Fleming”, Institute for Fundamental Biomedical Research, Vari, Greece; ^2^1st Department of Neurology, Memory and Movement Disorders Clinic, Eginition Hospital, Medical School, National and Kapodistrian University of Athens, Athens, Greece

**Keywords:** Tau, Tau phosphorylation, toxicity, neuronal dysfunction, learning deficits, gatekeeper phosphorylation, Drosophila

## Abstract

Hyperphosphorylated Tau protein is the main component of the neurofibrillary tangles, characterizing degenerating neurons in Alzheimer’s disease and other Tauopathies. Expression of human Tau protein in Drosophila CNS results in increased toxicity, premature mortality and learning and memory deficits. Herein we use novel transgenic lines to investigate the contribution of specific phosphorylation sites previously implicated in Tau toxicity. These three different sites, Ser^238^, Thr^245^, and Ser^262^ were tested either by blocking their phosphorylation, by Ser/Thr to Ala substitution, or pseudophosphorylation, by changing Ser/Thr to Glu. We validate the hypothesis that phosphorylation at Ser^262^ is necessary for Tau-dependent learning deficits and a “facilitatory gatekeeper” to Ser^238^ occupation, which is linked to Tau toxicity. Importantly we reveal that phosphorylation at Thr^245^ acts as a “suppressive gatekeeper”, preventing phosphorylation of many sites including Ser^262^ and consequently of Ser^238^. Therefore, we elucidate novel interactions among phosphosites central to Tau mediated neuronal dysfunction and toxicity, likely driven by phosphorylation-dependent conformational plasticity.

## Introduction

Tau is a multifunctional neuronal microtubule-associated protein (Sotiropoulos et al., 2017) important for regulation of axonal transport and the length of the labile domains of axonal microtubules (Qiang et al., 2018). A single gene gives rise to 6 protein isoforms in the adult human brain via alternative splicing that results in isoforms that contain 1, 2 or none sequence blocks at their amino-terminus (0N, 1N, 2N), as well as inclusion of exon 10, or its exclusion from the mRNA (Andreadis et al., 1992). This leads to isoforms with 3 (3R isoforms), or 4 (4R isoforms) caboxy-terminal repeats, which mediate interactions with the microtubular cytoskeleton (Buee et al., 2000). A predominant characteristic of all 6 Tau isoforms present in the human brain is their highly elevated steady state phosphorylation load mediated by the opposing actions of several kinases and phosphatases (Johnson and Stoothoff, 2004). Tau has 84 putative phosphorylation sites and it is reported more extensively phosphorylated during development than in mature neurons (Brion et al., 1993; Yu et al., 2009). Multiple kinases have been shown to phosphorylate Tau *in vitro* and in cells, but whether they also target Tau *in vivo* and under what circumstances remains largely elusive (Hosoi et al., 1995; Hong et al., 1997; Zheng-Fischhofer et al., 1998; Gong et al., 2005).

Interestingly, studies indicate that Tau phosphorylation might be primed by occupation of a particular site before the occurrence of additional phosphorylations (Hanger et al., 2009). Tau contains multiple “intrinsically disordered regions” (IDRs), which interfere with structural stability of the protein (Uversky, 2015). Then, this “gatekeeper” phosphorylation effect suggests that it may enable or inhibit local tertiary structures that expose or occlude other, often distant, phosphorylation sites (Jeganathan et al., 2008; Sibille et al., 2012; Schwalbe et al., 2015). Hence, the effect of specific phosphorylations may be to regulate this “structural plasticity” of Tau, contribute to the subcellular localization of Tau isoforms (Sotiropoulos et al., 2017) and modulate their functional properties (Xia et al., 2015).

Irrespective of whether it is mutated, or wild-type, pathological Tau presents increased phosphorylation at sites occupied physiologically, but also on sites occupied only when pathology is present and are referred to as “disease-associated” epitopes (Morris et al., 2015; Arendt et al., 2016). Although the mechanisms that trigger hyper-phosphorylation are unclear at present, the result is neuronal deposition of hyper-phosphorylated Tau (Martin et al., 2011). If indeed phosphorylations modulate the structure and functional properties of Tau isoforms, then this hyper-phosphorylation is likely to underlie significant changes in the properties of the protein that underlie its pathobiology (Regan et al., 2017). In fact, extensive literature has led to the widely held notion that aberrant Tau phosphorylation is central to neuronal pathology (Stoothoff and Johnson, 2005) and provided evidence that soluble hyper-phosphorylated Tau contributes to neuronal dysfunction before its aggregation (Fath et al., 2002; Santacruz et al., 2005; Brandt et al., 2009; Decker et al., 2015).

Antibodies that recognize non-physiologically phosphorylated Tau at specific sites (phosphoepitopes) in patient neurons but not in age-matched healthy individuals have been developed and used as specific diagnostic markers of Tauopathies (Sergeant et al., 2005). However, the mechanistic understanding of the sequential phosphorylation events that occur on Tau and which sites are essential for maintenance and evolution of pathology are still unclear. Identification of phosphorylation sites on Tau that either trigger or are essential for pathogenesis are pivotal to our understanding of Tau-dependent neuronal malfunction and toxicity.

Drosophila models of Tauopathies contribute significantly to the concept that accumulation of prefibrillar hyper-phosphorylated forms of Tau correlate with human Tau-mediated toxicity in flies (Wittmann et al., 2001; Steinhilb et al., 2007a, b; Feuillette et al., 2010). Recently, we have identified two novel phosphorylation sites on Tau, Ser^238^ and Thr^245^, as essential for its toxic effects on mushroom body (MB) integrity (Kosmidis et al., 2010; Papanikolopoulou et al., 2010) and premature lethality (Papanikolopoulou and Skoulakis, 2015). The MBs are neuronal assemblies that constitute major insect brain centers for learning and memory (Davis, 2005). Significantly, blocking Ser^238^ and Thr^245^ phosphorylation by substituting them with alanines (STA mutant), yielded animals with structurally normal but profoundly dysfunctional MBs, as flies accumulating the mutant protein exhibited impaired associative learning (Kosmidis et al., 2010). Moreover, our results strongly suggested that Ser^238^ occupation is a critical mediator of Tau neurotoxicity *in vivo*. However, neuronal dysfunction measured as olfactory learning deficits appeared to rely on phosphorylation at Ser^262^, whose enhanced occupation appeared to account for the learning deficits of animals expressing the STA mutant Tau (Papanikolopoulou and Skoulakis, 2015).

Our previous analyses were performed on the double mutant STA and with Tau transgenes in different genomic locations with consequent variable transgenic protein expression, which attenuated accurate quantitative measures of toxicity and dysfunction and their dependence on relative hTau levels in the CNS. Therefore, we aimed to investigate the contribution of each of these two sites independently and investigate the potential interplay between them with respect to neuronal toxicity and dysfunction with quantifiably equally expressed transgenes. Hence, we generated new single mutants that block or mimic phosphorylation at these sites. We also focus on the role of Ser^262^ as a “gatekeeper” of disease-associated phospho-epitope appearance and whether as previously suggested (Papanikolopoulou and Skoulakis, 2015), its occupation is regulated by phosphorylation of Ser^238^ or Thr^245^ or *vice versa*.

## Materials and Methods

### Drosophila Culture and Strains

Drosophila crosses were set up *en masse* in standard wheat-flour-sugar food supplemented with soy flour and CaCl_2_ and cultured at 25°C and 50–70% humidity in a 12 h light/dark cycle unless noted otherwise. The Elav^c155^-Gal4 and Ras2-Gal4 have been described before (Gouzi et al., 2011). The Elav-Gal4 line on the second chromosome was obtained from Bloomington Drosophila Stock center (#8765). The dual Gal 4 driver strains Elav^c155^-Gal4;Ras2-Gal4 (henceforth Elav;Ras2) and Elav^c155^-Gal4;Elav/CyO (henceforth Elav;Elav), were constructed by standard crosses. The hTau^0N4*R*^ (0N4R) transgenic flies were a gift from M. Feany (Harvard Medical School, Boston, MA, United States).

To generate the transgenics carrying point mutations, the hTau^0N4*R*^ cDNA (kind gift from Dr. Martin Chow, University of Kentucky), was subcloned into the pUAS.attB vector (Bischof et al., 2007) as a *Bgl*II*/Xba*I fragment. The mutants were generated by replacing the indicated Ser and Thr residues with Ala or Glu using the QuickChange XL site-directed mutagenesis kit (Agilent) according to the manufacturer’s instructions. The complementary mutagenic oligonucleotides pairs (5’ to 3’) for each mutant are shown below. The silent restriction sites introduced for effective screening of positive clones appear underlined in italics whereas the amino acid substitution is shown in bold.

S238A (*Pvu*II): 5’ CCACCCAAGTCGCCGT*CA* ***GCT****G*CCAAGAGCCGCCTGCAGACAGC.

GGTGGGTTCAGCGGCAG*T****CGA****C*GGTTCTCGGCG GACGTCTGTCG 5’.

S238E (*Xba*I): 5’ CCAAGTCGCCGTCT**GAG**GCCAAG *TCTAGA*CTGCAGACAGCCCC.

GGTTCAGCGGCAGA**CTC**CGGTTC*AGATCT*GACGTCTG TCGGGG 5’.

T245A (*Sal*I): 5’ CTTCCGCCAAGA*GTCGAC*TGCAG**GCA**G CCCCCGTGCCCATG.

GAAGGCGGTTCT*CAGCTG*ACGTC**CGT**CGGGG GCACGGGTAC 5’

T245E (*Sal*I): 5’ CGTCTTCCGCCAAGA*GTCGAC*TGCAG **GAG**GCCCCCGTGCCCATG.

GCAGAAGGCGGTTCT*CAGCTG*ACGTC**CTC**CGGGGG CACGGGTAC 5’.

S262A (*Bgl*II): 5’ GCC*AGATCT*GAAGAATGTCAAGTCCAA GATCGGC**GCC**ACTGAG.

CGG*TCTAGA*CTTCTTACAGTTCAGGTTCTAGCCG**CGG** TGACTC 5’.

S262E (*Bgl*II): 5’ GCC*AGATCT*GAAGAATGTCAA GTCCAAGATCGGC**GAG**ACTGAG.

CGG*TCTAGA*CTTCTTACAGTTCAGGTTCT AGCCG**CTC**TGACTC 5’.

STA (*Pvu*II): 5’ CCAAGTCGCCGT*CA****GCT****G*CCAAGAGCC GCCTGCAG**GCA**GCCCCCG.

GGTTCAGCGGCA*GT****CGA****C* GGTTCTCGGCGGACGTC**CGT**CGGGGGC 5’.

The sequence of the mutants was confirmed by sequencing (VBC-biotech). Transgenic flies were generated by phiC31-mediated transgenesis by BestGene Inc. (Chino Hills, CA, United States). DNAs were injected into genomic landing site 53B2 on the second chromosome (BDSC #9736).

To obtain flies for experiments detailed herein, virgins from the driver strains were crossed to males bearing the hTau transgenes and experimental genotypes were selected from the progeny as appropriate and as detailed below:

Female Elav X 0N4R or 0N4R^II^ or w^1118^ (for controls).

Female Elav;Elav/CyO X 0N4R or 0N4R^II^: Select male and female non-CyO progeny.

Female Elav;Ras2 × 0N4R or 0N4R^II^: Select male and female non-CyO progeny.

Female Elav;Ras2 × 0N4R^S238A^ or 0N4R^S238E^ or 0N4R^T245A^ or 0N4R^T245E^ or 0N4R^S262A^ or 0N4R^S262E^ or 0N4R^STA^: Select male and female progeny.

Female Elav;Ras2 X w^1118^: Select male and female progeny for controls.

### Histology

Immunohistochemistry on paraffin sections of Drosophila adult heads were performed on paraffin sections as previously described (Kosmidis et al., 2010) using the anti-Leo primary antibody at 1:4000.

### Western Blot and Antibodies

Total Tau levels and occupation of particular phosphosites were determined in 2–5 adult female head homogenates in 1x Laemmli buffer (50 mM Tris pH 6.8, 5% 2-mercaptoethanol, 2% SDS, 10% glycerol, and 0.01% bromophenol blue). The monoclonal antibodies T46 (recognizing hTau irrespective of phosphorylation- Invitrogen, 1:3,000), AT8 (recognizing phosphorylated Ser^202^/Thr^205^-Pierce Endogen, 1:1,000), AT100 (recognizing phosphorylated Thr^212^/Ser^214^-Pierce Endogen 1:1,000) and the polyclonal antibodies pS396 (Source Bioscience, 1:3,000) and pS262 (Source Bioscience, 1:1,000) were used. We generated a novel polyclonal antibody against phospho-Ser^238^ of hTau, raised in rabbits (Pocono Rabbit Farm and Laboratory) against peptide TPPKSPS**p**SAKSRLQTAPVPMP and affinity purified before use. In order to normalize sample loading, anti-Syntaxin (mAb 8C3, Developmental Studies Hybridoma Studies) was used at 1:3,000. Secondary antibodies were applied at 1:5,000.

### Lifespan Determination

Flies accumulating hTau^0N4R^ variants under Elav^c155^-Gal4;Ras2-Gal4 were raised at 18°C and subsequently 17 groups of 20 (340 flies per genotype in two biological replicates), 1–2 day-old males per genotype were maintained at 30°C transferring to fresh vials every 3 days until they expired (Papanikolopoulou and Skoulakis, 2015).

### Drug Feeding

Flies accumulating hTau^0N4R^ variants under Elav^c155^-Gal4; Ras2-Gal4 were raised at 25°C together with control heterozygotes. Paraquat feeding was performed as previously described (Dias-Santagata et al., 2007). Briefly fifteen groups of 20, (two biological replicates of 300 flies total) 1–2 day-old males per genotype were fed 30 mM of methyl viologen (Acros Organics) in standard food for 24 h or as indicated and the number of surviving flies per vial were counted.

### Behavioral Assessment

Behavioral assays were performed under dim red light at 24–25°C and 70-75% humidity. All flies were 2–5 days old, collected under CO_2_ anesthesia one day prior to the experiment and kept in food vials in groups of 50–70 flies each at 30°C. Pavlovian olfactory aversive conditioning was performed using aversive odors as conditioned stimuli (CS+ and CS−) with the electric shock unconditioned stimulus (US). The odors used were benzaldehyde (BNZ) and 3-octanol (OCT) diluted in oil (5% v/v for BNZ and 50% v/v for OCT). One hour before training flies were transferred to fresh food vials. For training, a group of 50–70 flies were transferred into a tube lined with electrifiable grid and presented with air (500 mL/min). Flies were first exposed to an odor for 30 s paired with 90 V shock (consisting of six 1.25 s pulses with 4.5 s inter-pulse intervals, so 6 US/CS pairings were delivered within 30 s of odor presentation) and then 30 s of air. Subsequently, flies were exposed to the second odor for 30 s without shock and then 30 s of air. Two groups of animals of the same genotype were trained simultaneously such as, one to avoid BNZ and the other to avoid OCT, while the complimentary odorant was used as control odor. The animals were transferred to a T-maze apparatus immediately and flies were tested simultaneously for preferential avoidance of the conditioned odorant allowed to choose between the two odors for 90 s. A performance index (PI) was calculated as the fraction of flies that avoided the CS+ minus the fraction that avoided the CS− odors divided by the total number of flies in the experiment. A final PI is the average of the scores from the two groups of flies trained to complementary conditioning stimuli and ranges from 0 to 100. Because all new transgenes are inserted in the same site we used one of the cognates as heterozygous controls.

### Statistical Analyses

Quantification of all Western blots was performed by densitometry and the ratio of Tau relative to that of Syntaxin (Syx) was calculated. The ratio of the control genotype was set to 1 and all experimental ratios were reported as relative to that. The means and SEMs were compared following an initial, significant differences- indicating ANOVA (positive ANOVA), using Dunnett’s tests relative to the designated control. Similarly, learning performance indices were calculated for each genotype as indicated above and following positive ANOVA, means and SEMs were compared to that of controls using Dunnett’s tests.

Survival data were examined for differences at each assessment day using Wilcoxon/Kruskal-Wallis tests. If significant differences were uncovered then the means and SEMs from each genotype for that day were compared relative to controls using the Steel with control tests. Means and SEMs of survival upon oxidative stress toxicity were compared to that that of controls or the variants as designated using Dunnett’s or planned multiple comparisons tests following an initial positive ANOVA. All statistical details are presented in Supplementary Tables 1–5.

## Results

### Learning Deficits Are Independent of hTau Levels in Adult Drosophila

Because the randomly inserted hTau^STA^ was generated in the 2N4R isoform (Kosmidis et al., 2010), to generalize the effects of that double mutation and validate the derived conclusions (Papanikolopoulou and Skoulakis, 2015) independently, we generated the STA double mutation in the 0N4R isoform. In addition, comparison of the effects of transgenics on neuronal function and neurotoxicity is expected to be facilitated if the protein levels were similar or the same, rather than the variable levels attained by randomly inserted transgenes. Therefore, we generated new 0N4R, STA and single phosphomutant strains via phiC31-mediated transgenesis (Bischof et al., 2007), with all transposons inserted in the same landing site on the second chromosome (55B2), expected to yield similar expression levels to facilitate cross variant comparisons. Importantly, all new transgenes used herein presented similar levels of expression (Supplementary Figure 1), ascertaining that potential phenotypic differences among the transgenic proteins would not be due to differences in expression, but rather reflect differential functional effects.

**FIGURE 1:**
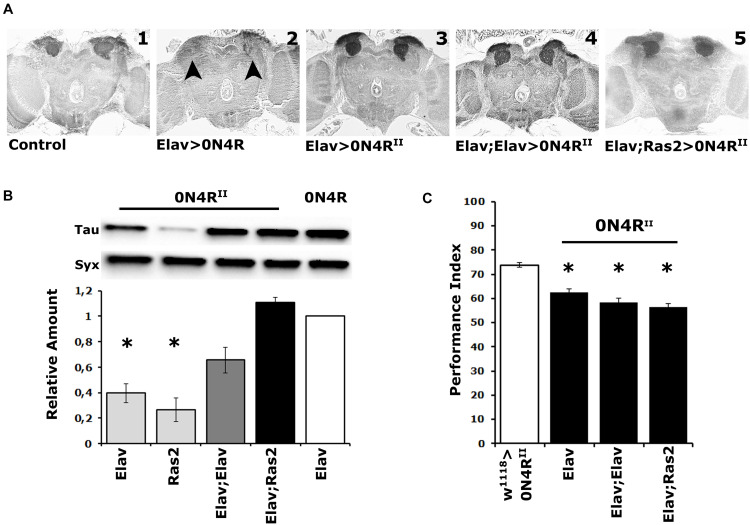
Elevation of hTau in the fly CNS does not precipitate MB structural deficits, and the MBs remain dysfunctional over a range of transgenic protein accumulation. **(A)** Carnoy’s-fixed paraffin-embedded frontal sections at the level of the MB dendrites (calyces) from control (Elav/ +) and animals expressing the different 0N4R-encoding transgenes with the indicated Gal4 driver. The morphology of the MBs was evaluated with the anti-Leonardo antibody. Arrowheads point to the missing calyces in animals expressing the randomly inserted 0N4R human transgene. **(B)** Representative Western blot of head lysates from animals expressing the 0N4R^II^ and 0N4R transgenes under the indicated Gal4 driver using the T46 anti-Tau antibody. The genotype of animals used is indicated below the quantification. For the quantification, Tau levels were normalized using the Syx loading control and are shown as a ratio of their mean ± SEM values relative to respective levels in Elav > 0N4R flies, which was set to 1. The star indicates significant differences from the control genotype indicative of reduced Tau levels. Statistical details in Supplementary Table 1. **(C)** Learning performance of animals accumulating pan-neuronally the indicated 0N4R transgenes (black bars), compared with transgene heterozygotes (white bars). The genotypes of all animals are indicated below each bar. The means ± standard error of the mean (SEM) are shown. Stars indicate significant differences from control. Statistical details in Supplementary Table 1.

Interestingly, unlike for the randomly inserted hTau^0N4R^ (henceforth 0N4R) (Wittmann et al., 2001), pan-neuronal expression of the same transgene integrated in the second chromosome landing site (0N4R^II^) did not result in MB ablation (Figure 1A2 vs. Figure 1A3), as previously reported (Kosmidis et al., 2010). We hypothesized that integration within the attp sites, or at the specific location on the second chromosome must have attenuated 0N4R^II^ expression resulting in intact MBs. To address this and because all phosphosite variants we generated were integrated in the same chromosomal site, we sought to increase expression of these transgenes to match that of 0N4R. To that end we combined two copies of the pan-neuronal Elav driver, the typical one on the X chromosome with an independent insertion on the second (Elav;Elav), or the pan-neuronal Elav driver on the X with the ubiquitous adult CNS driver Ras2 (Gouzi et al., 2011) as detailed in Materials and Methods.

Quantification of 0N4R^II^ levels relative to those of the original 0N4R revealed significant differences under Elav, Ras2, but not the double driver Elav;Elav, or Elav;Ras2 (Figure 1B and Supplementary Table 1). Importantly, 0N4R^II^ accumulation under Elav;Ras2 was the closest to the levels of 0N4R under Elav (Figure 1B and Supplementary Table 1) as reported before (Kosmidis et al., 2010). Therefore, the Elav;Ras2 composite driver was used for all subsequent experiments. Interestingly, however, despite the similar levels of expression the MBs remained structurally intact in animals expressing 0N4R^II^ under Elav;Ras2 (Figure 1A5), as they also did under Elav;Elav (Figure 1A4). In fact quantification of the dendritic areas (calyces) stained by the anti-Leo antibody in controls (0.1376 mm^2^) compared to those in animals expressing 0N4R^II^ under Elav;Ras2 (0.1268 mm^2^) did not yield significant differences (Student’s *t*-test, *p* = 0.1716, *n* = 6). This indicates that the structural deficits of the MBs are highly sensitive to the amount of hTau present during their development (Kosmidis et al., 2010) and levels under Elav;Ras2 are likely just under the threshold requisite to precipitate defects. Alternatively, the presence of the attp sites flanking the new transgenes may insulate them from non-specific effects of neighboring chromatin on their levels, or temporal expression pattern. This likely leads to bypassing the sensitive period during early embryogenesis when hTau elevation results in aberrant MBs (Kosmidis et al., 2010).

Importantly, expression of the 0N4R^II^ transgene under the single Elav, the Elav;Elav or the Elav;Ras2 drivers yielded similar deficits in associative learning (Figure 1C and Supplementary Table 1). Although the lowest performance index was consistently attained under Elav;Ras2, it was marginally (p = 0.002) different than that under Elav and not different from the performance of animals expressing 0N4R^II^ under Elav;Elav (Supplementary Table 1). Since 0N4R^II^ expression under all pan-neuronal drivers tested yielded similar learning deficits, these results demonstrate that the magnitude of learning impairment is not proportional to the levels of hTau and is not consequent of altered MB integrity. Therefore, we used Elav;Ras2 to drive 0N4R^II^ for subsequent analyses because it induces expression levels similar to previously used and reported conditions (Kosmidis et al., 2010; Papanikolopoulou et al., 2010; Papanikolopoulou and Skoulakis, 2015), which would facilitate comparisons and extrapolations.

### Differential Contributions of Ser^238^ and Thr^245^ to hTau-Dependent Toxicity

**FIGURE 2:**
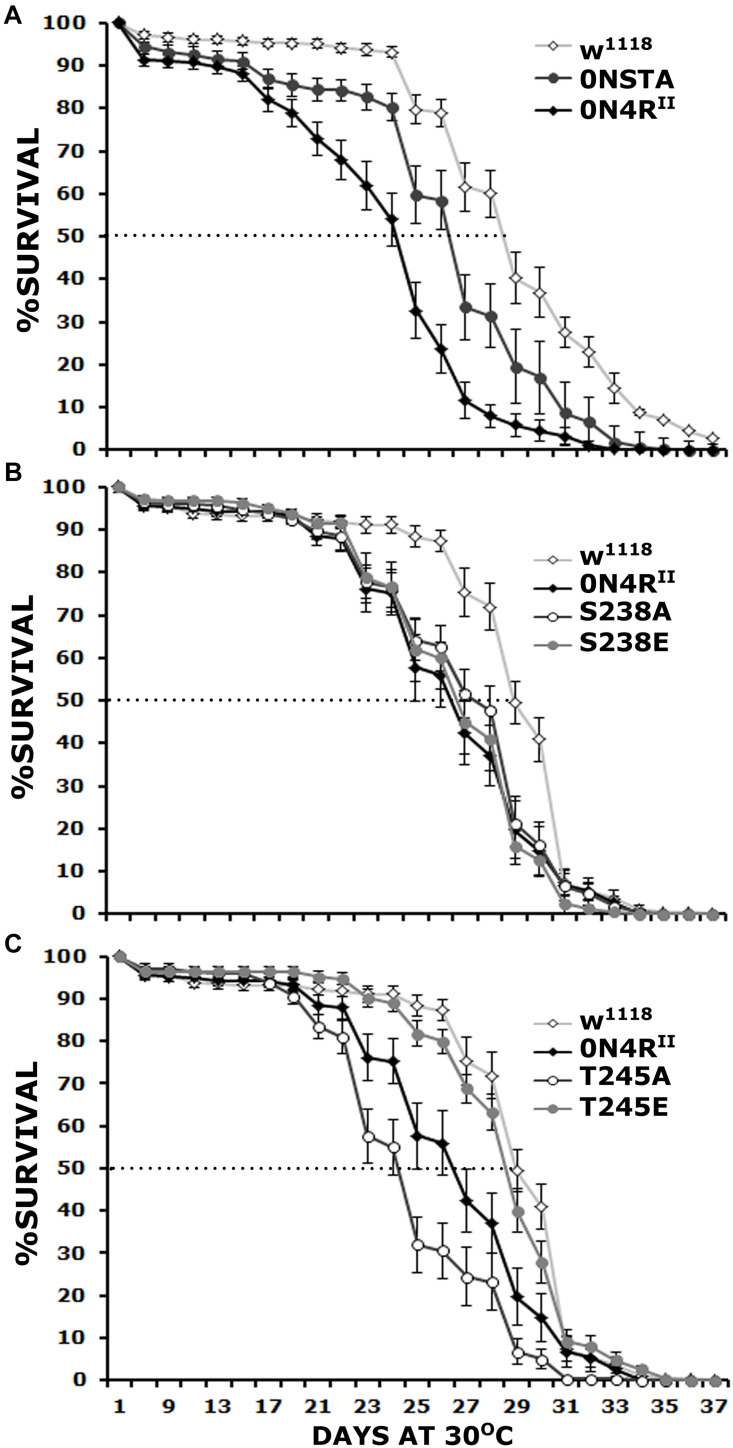
Differential effects of mutants on Ser^238^ and Thr^245^ on premature lethality. Survival curves for animals expressing 0N4R^II^, 0NSTA **(A)**, S238A, S238E **(B)**, T245A, and T245E variants **(C)** expressed in the CNS in comparison with Elav/+; Ras/ + control heterozygotes (w^1118^). The data represent the mean ± SEM from two independent experiments with a total of 340 flies assessed per genotype. After 29 days, the population of Elav/+; Ras/ + control heterozygotes (w1118) was reduced by 50% (50% attrition). Statistical details in Supplementary Table 2.

Initially we determined whether the Ser^238^ and Thr^245^ to Ala double mutation (STA), on the 0N4R isoform suppresses hTau toxicity measured as elevated age-dependent mortality as did when on the 2N4R isoform (Kosmidis et al., 2010). To that end, we raised animals at 18C to keep transgene expression minimal and then transferred and maintained the adults to 30C to maximize transgene expression and scored for survivors every 2 days. Importantly, expression of 0N4R^II^ resulted in premature lethality relative to controls (Figure 2A and Supplementary Table 2), similar to the randomly inserted transgene (Papanikolopoulou and Skoulakis, 2015). In contrast, expression of 0N4R^STA^ did not yield significantly different survival profile from that of controls (Figure 2A and Supplementary Table 2). Significantly, on the 29th day when the population of control animals is reduced by 50% (50% attrition-dotted line on Figure 2A), the population of surviving 0N4R^II^ animals was significantly different from controls while that of 0N4R^STA^-expressing flies was not (Supplementary Table 2). Therefore, the new transgenes on chromosome II fully recapitulate and verify the survival results obtained with the randomly inserted transgenic strains (Papanikolopoulou and Skoulakis, 2015). Because the new transgenes are expressed at the same level (Supplementary Figure 1), these results confirm that toxicity is independent of hTau protein levels and the assertion that Ser^238^ or Thr^245^, or both are required for hTau dependent toxicity manifested as premature mortality.

To determine whether both, or which of the two phosphorylation sites mutated in the STA transgene mediate hTau toxicity, we examined the effects of single substitutions of Ser^238^ and Thr^245^ either to the phosphorylation-blocking Ala, or the potential phosphomimic Glu. Ser^238^ has been reported phosphorylated immediately before the 50% attrition point on 0N4R (Papanikolopoulou and Skoulakis, 2015), leading to the hypothesis that its occupation predicts toxicity. Surprisingly, however, blocking phosphorylation at Ser^238^ did not ameliorate the toxicity of the 0N4R^S238A^ protein (Figure 2B), which remained significantly different from that of control flies from days 23 until 30, including day 29 when the control population reached the 50% attrition day (Figure 2B and Supplementary Table 2). The pseudophosphorylated 0N4R^S238E^ also remained as toxic as the parental 0N4R^II^ (Figure 2B and Supplementary Table 2). The results suggest that phosphorylation at Ser^238^ may be necessary, but it is not sufficient to induce of hTau toxicity underlying early mortality, unlike our prior hypothesis (Papanikolopoulou and Skoulakis, 2015).

Significantly, the 0N4R^T245A^ protein where Thr^245^ phosphorylation was blocked by the Ala substitution presented consistently elevated mortality from days 23 to 30 (Figure 2C and Supplementary Table 2), earlier than in animals expressing 0N4R^II^ and presenting a 50% attrition on day 24 instead of day 29 in controls (Figure 2C). This suggests that occupation of this site is essential to suppress toxicity leading to early mortality. Surprisingly and in accord with this hypothesis, the phosphomimic 0N4R^T245E^ mutation ameliorated toxicity behaving much like controls throughout the curve including the 50% attrition day (Figure 2C and Supplementary Table 2). These results strongly suggest that phosphorylation at Thr^245^ is protective or averts toxicity, whereas lack of occupation at that site is necessary for hTau toxicity linked to early mortality.

**FIGURE 3:**
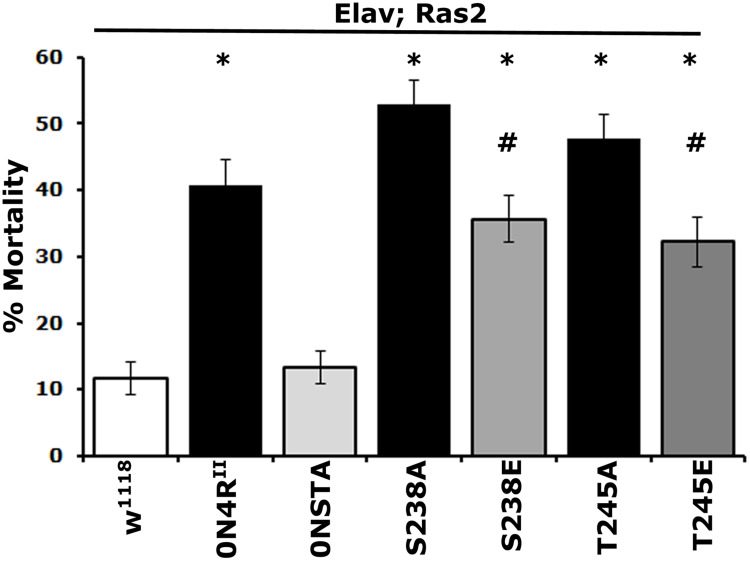
Differential effects of mutants on Ser^238^ and Thr^245^ on oxidative stress. One to 2-day-old flies were exposed to 30 mM paraquat, and mortality was scored after 24 h. The data represent the mean ± SEM from two independent experiments with a total of 300 flies assessed per genotype. The genotypes of all animals are indicated below each bar. Stars indicate significant difference from controls and **^#^** significant difference from the cognate mutant. Compared with the control driver heterozygote flies (Elav/+; Ras/+) administration of paraquat for 24 h resulted in significant lethality for all Tau variants except for 0NSTA expressing flies. Statistical details in Supplementary Table 3.

A different measure of toxicity that underlies the level of oxidative stress upon pathological hTau accumulation is resistance to paraquat. This is a redox-active bipyridine heterocyclic compound, which in tissues produces superoxide anions and therefore exacerbates already extant oxidative stress (Rzezniczak et al., 2011). Consequently, flies experiencing increased oxidative stress due to pathological hTau accumulation are expected to be more sensitive to added paraquat-mediated stress (Dias-Santagata et al., 2007). In fact, after 24 h of exposure to 30 mM paraquat-laced food, control flies presented a baseline 10% mortality, while 40% of 0N4R^II^ animals expired in the same time frame. In contrast, the Ser^238^ and Thr^245^ to Ala double mutation 0N4R^*STA*^-expressing animals suppressed mortality to basal levels similar to that of controls (Figure 3 and Supplementary Table 3), suggesting a role for both Ser^238^ and/or Thr^245^ in hTau-mediated oxidative stress toxicity.

As for mortality, the 0N4R^S238A^ protein with blocked phosphorylation at Ser^238^ and the pseudophosphorylated 0N4R^S238E^ were as toxic as 0N4R^II^ (Figure 3 and Supplementary Table 3). However, toxicity of 0N4R^S238E^ was significantly milder than that of 0N4R^S238A^ (Supplementary Table 3). Blocking Thr^245^ phosphorylation presented elevated hTau toxicity compared to 0N4R^II^-expressing animals (Figure 3 and Supplementary Table 3). Interestingly 0N4R^T245A^ was ameliorated to 0N4R^II^ levels by pseudophophorylation at that site, but it was not eliminated to 0N4R^STA^ basal levels (Figure 3 and Supplementary Table 3). Because expression of the double phosphoblock mutant 0N4R^STA^ presented only baseline toxicity unlike the single site mutants (Supplementary Table 3), these results suggest that both sites likely need to be occluded from phosphorylation to eliminate or suppress hTau-mediated oxidative stress toxicity. The dependence of oxidative stress-dependent lethality on occupation of Ser^238^ and Thr^245^ is distinctly different from that for premature mortality, suggesting that the two measures of toxicity may in fact reflect distinct pathological mechanisms.

### Multiple Phosphorylations Depend on the State of Thr^245^ and Ser^238^ Occupation

**FIGURE 4:**
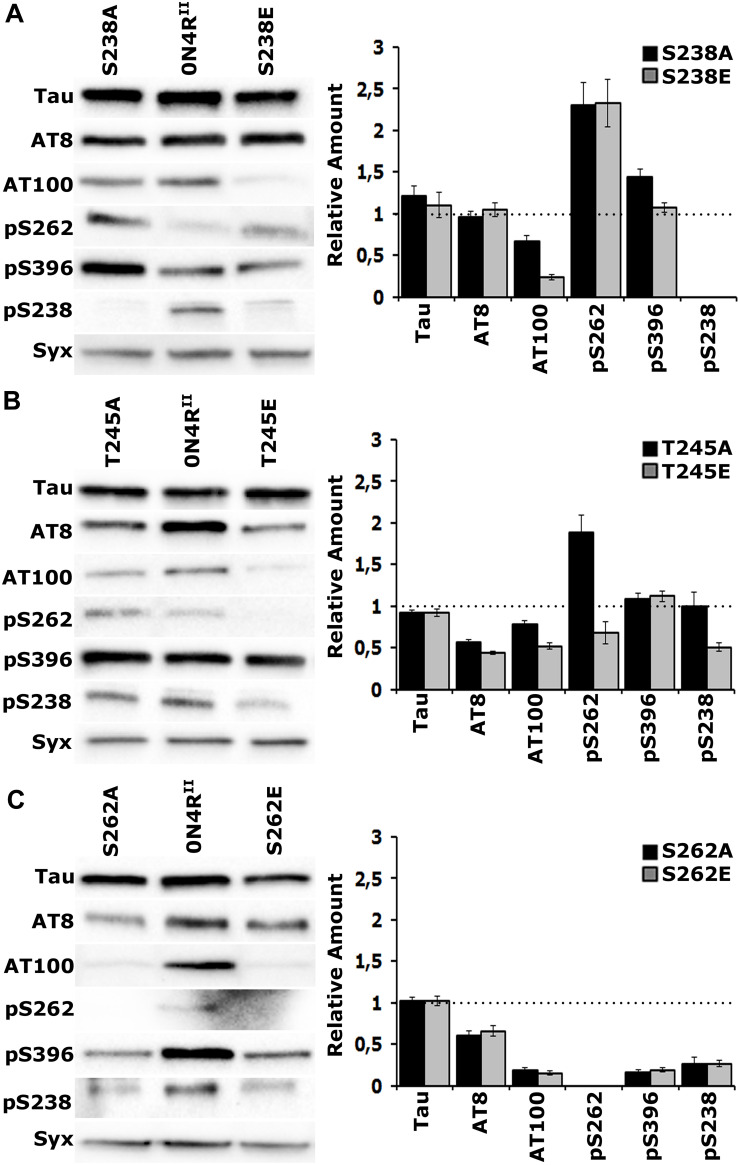
Gatekeeper phosphorylation interactions among Ser^238^, Thr^245^, and Ser^262^. Representative Western blots from head lysates of flies accumulating 0N4R^II^ pan-neuronally compared with similar lysates from site-specific Ala and Glu mutants probed with the antibodies indicated on the left. The level of syntaxin (Syx) in the lysates was used as loading control for quantifications. The first antibody measures total Tau in the lysates, whereas all others target particular phosphorylated residues. Quantification of at least three independent biological replicates of at least six technical replicates are shown on the right. The syntaxin-normalized level of 0N4R^II^ for each quantification was fixed to 1 and represented by the horizontal dotted line. The bars represent the mean ± SEM relative levels of mutants phosphorylated at the given sites, over that of the 0N4R^II^ control. Bars and their respective error bars that are not in contact with the dotted line are significantly different from control. Statistical details in Supplementary Table 4. **(A–C)** Representative Western blots from head lysates of flies accumulating 0N4R^II^ pan-neuronally compared with similar lysates from S238A and S238E mutants **(A)**, T245A and T245E mutants **(B)** and S262A and S262E mutants **(C)** probed with the antibodies indicated on the left. Dunnett’s tests followed initial ANOVA to indicate significant differences from control for all phosphosites.

To investigate the proposed interaction between occupation of Ser^238^ and Thr^245^ and its consequences on distant sites, we used phospho-specific antibodies against sites associated with pathology. We selected the AT8 epitope formed by phosphorylation of Ser^202^ and Thr^205^ and AT100 signifying occupation of Thr^212^ and Ser^214^ in the Proline Rich Region of hTau, amino-terminal to Ser^238^ and Thr^245^. In addition, we assayed phosphorylation at Ser^396^ near the carboxy-terminus of the protein. As demonstrated again on Figures 4A–C, the expression levels of the new transgenes are the same and equivalent with that of 0N4R^II^. In addition, we verified the specificity of the new anti-pSer^238^ antibody against 0N4R^STA^ (Supplementary Figure 2). A residual band of higher molecular mass was detectable in all lysates including 0N4R^STA^ (Supplementary Figure 2) and is considered non-specific.

Blocking phosphorylation, or pseudophosphorylation of Ser^238^ reduced AT100 phosphorylation (Figure 4A and Supplementary Table 4) and elevated occupation of Ser^262^ (Figure 4A and Supplementary Table 4) relative to that on 0N4R^II^. This suggests that Ser^262^ occupation is independent of the state of Ser^238^ phosphorylation. Phosphorylation of the distant Ser^396^ was elevated upon blockade of Ser^238^ occupation, but remained unaffected by pseudophosphorylation of that site (Figure 4A and Supplementary Table 4). As expected, the anti-pSer^238^ antibody also did not recognize the 0N4R^*S*238*A*^, or the 0N4R^*S*238*E*^ proteins (Figure 4A).

Importantly, although Ser^238^ occupation appeared unaffected in the 0N4R^T245A^ protein, it was significantly reduced in the pseudo-phosphorylated 0N4R^T245*E*^ (Figure 4B and Supplementary Table 4). This is consistent with the notion that occupation of Thr^245^ suppresses Ser^238^ phosphorylation. Given that Glu substitution provides a single charge at the site vs. the two charges carried by the phosphate group, the suppression may in fact be higher in the normal wild type hTau. Because an anti- pThr^245^ antibody is not currently available, we cannot currently directly test whether the reciprocal that is, Ser^238^ occupation suppressing Thr^245^ phosphorylation may be true as well. Conversely, blockade of Thr^245^ occupation enhanced Ser^262^ relative to that on 0N4R^II^, while pseudophosphorylation left it at control levels if not marginally lower (Figure 4B and Supplementary Table 4). This is consistent with the notion that loss or suppression of Thr^245^ phosphorylation leads to Ser^238^ and Ser^262^ occupation. Inasmuch as the phosphomimic substitution may not fully reflect the consequence of *bona fide* phosphorylation at the site, the data support the notion Thr^245^ occupation suppresses both Ser^238^ and Ser^262^ phosphorylation. Therefore, in effect it acts as a “gatekeeper” for the consequent pathological manifestations of Ser^238^ and Ser^262^ occupation (Papanikolopoulou and Skoulakis, 2015).

The proposed role of Thr^245^ as the critical “gatekeeper” instead of the Ser^238^, the two residues concurrently changed in the STA mutants is also reflected in the consequences of blocking, or pseudophosphorylating it on distant phosphosites. Phosphorylation at the proximal phospho epitopes recognized by the AT8 and AT100 antibodies are significantly depressed in both Thr^245^ mutants (Figure 4B and Supplementary Table 4), but Ser^396^ occupation remains unaffected. In contrast, only AT100 occupation is suppressed in Ser^238^ mutants, while Ser^396^ phosphorylation appears significantly elevated when Ser^238^ phosphorylation is blocked (Figure 4A and Supplementary Table 4). This suggests that Thr^245^ occupation is required for phosphorylation at the AT8 and AT100 epitopes, while Ser^238^ phosphorylation suppresses occupation of Ser^396^, another manifestation of potentially conformation-mediated interactions among distant phosphosites.

The essential role of Ser^262^ as a broad-acting “gatekeeper” of pathology (Papanikolopoulou and Skoulakis, 2015) in accord to independently-derived similar proposal (Nishimura et al., 2004) is demonstrated in Figure 4C where all phosphorylations in question are drastically suppressed on the 0N4R^S262A^ and 0N4R^S262E^ proteins. This includes pSer^238^, which drastically reduced, albeit not completely eliminated (Figure 4C and Supplementary Table 4). Although the effect of blocking Ser^262^ phosphorylation (0N4R^S262A^), supports the notion that its occupation is required for subsequent Ser^238^ phosphorylation, the opposite might be expected for the phospho-mimic 0N4R^S262E^. The fact that Ser^238^ is under-phosphorylated on the 0N4R^S262E^ protein argues that the single charge- bearing Glu is not an efficient substitute for phosphorylated Ser^262^ in mediating conformational changes promoting phosphorylation at proximal and distant sites (Figure 4C). Rather it appears that it may in fact have a negative effect by occluding *bona fide* phosphorylation at that site.

### Differential Contribution of Ser^238^ and Thr^245^ to hTau-Mediated Learning Deficits

In addition to its “gatekeeper” effects, phosphorylation at Ser^262^ was proposed to be necessary for and predict learning deficits (Papanikolopoulou and Skoulakis, 2015). The Ser^262^ variants were in fact generated for a second reason in addition to facilitating comparison and validation of the effects of the Ser^238^ and Thr^245^ variants. Because the expression level of the previously used randomly inserted Ser^262A^ variant was low (Papanikolopoulou and Skoulakis, 2015), it raised the possibility that the lack of learning deficits upon expression of that transgene may have been consequent of its low expression.

**FIGURE 5:**
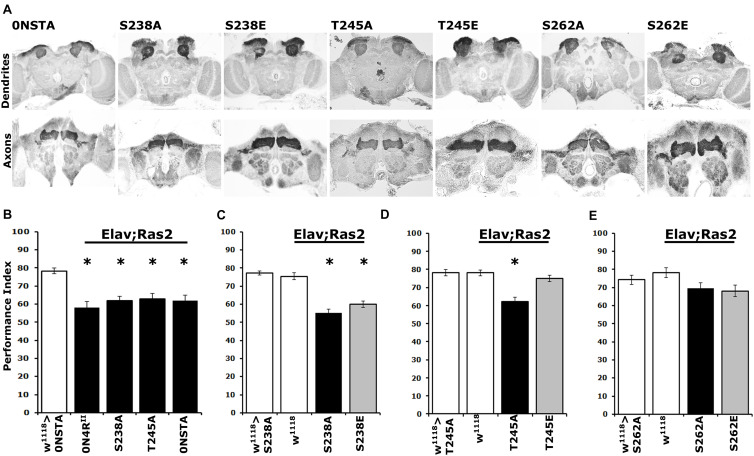
Differential learning defects of mutants on Ser^238^ and Thr^245^ and Ser^262^. **(A)** Carnoy’s-fixed paraffin-embedded frontal sections stained with anti-Leonardo at the level of MB dendrites in the posterior of the head and of MB axons of the γ-subtype-lobe from animals expressing the indicated Tau variants. **(B–E)** Learning performance of animals accumulating pan-neuronally the indicated Tau variants (black bars) compared with driver or transgene heterozygotes (white bar). The genotypes of all animals are indicated below each bar. The means ± standard error of the mean (SEM) are shown. Stars indicate significant differences from control. Statistical details in Supplementary Table 5.

Pan-neuronal expression of the new 0N4R^STA^ variant did not precipitate structural defects in the MBs (Figure 5A), in agreement with prior reports (Kosmidis et al., 2010; Papanikolopoulou and Skoulakis, 2015). Moreover, as for the 2N4R^STA^ (Kosmidis et al., 2010), expression of 0N4R^STA^ yielded significant deficits in associative learning (Figure 5B and Supplementary Table 5), equivalent to those presented by 0N4R^II^ (Supplementary Table 5), demonstrating that although grossly intact structurally, the CNS of animals expressing this hTau variant is dysfunctional. Importantly, expression of the single variants 0N4R^S238A^ and 0N4R^T245A^ resulted in a similar decrease in learning. Given that Ser^262^ is hyper-phosphorylated in these variants (Figures 4A,B), the data support the proposal that elevated occupation at this site is in fact requisite for the learning deficits (Papanikolopoulou and Skoulakis, 2015).

Further toward this notion, we assayed associative learning in animals expressing the phosphomimic variants 0N4R^S238E^ and 0N4R^T245E^ alongside the respective Ala substituted proteins at these sites. Interestingly, expression of 0N4R^S238E^ precipitated a learning deficit equivalent to that of the phospho-blocked variant 0N4R^S238A^ (Figure 5C and Supplementary Table 5), as predicted by the hyper-phosphorylation of this protein at Ser^262^ (Figure 4A) and in agreement with the inefficiency of Glu substitution as a phospho-mimic at that site. In contrast, unlike the deficit presented by animals expressing 0N4R^T245A^, expression of the pseudo-phosphorylated variant 0N4R^T245E^ did not affect learning (Figure 5D and Supplementary Table 5). This agrees with the phosphorylation state of Ser^262^ on this variant protein and in support of the proposed role of Thr^245^ phosphorylation as suppressive of hTau-mediated toxicity and dysfunction. This in turn suggests that phosphorylation at this epitope may be constitutive on non-pathogenic hTau.

As expected, expression of the phosphoblocked 0N4R^S262A^ protein did not affect learning (Figure 5E and Supplementary Table 5), confirming published results (Papanikolopoulou and Skoulakis, 2015). Moreover, since 0N4R^S262A^ is expressed at the same level as controls (Supplementary Figure 1 and Figure 4C), this result verifies that lack of deficient learning was not consequent of lower expression of the randomly inserted transgene (Papanikolopoulou and Skoulakis, 2015). Again, lack of the expected learning deficit upon expression of the pseudo-phosphorylated 0N4R^S262E^ is a likely consequence of the inefficient Glu-mediated mimic of phosphorylation at that site (Figure 5E).

## Discussion

Although hTau phosphorylation and its regulation has received waning attention lately, hyper-phosphorylation at disease linked phosphoepitopes remain strong pathology-linked biomarkers (Blennow and Zetterberg, 2018). Therefore, understanding the patterns and regulation of hTau phosphorylation is essential to monitor pathologies and their progression, but just as importantly, to understand its contribution to the IDR-mediated structural plasticity of this protein. The results herein describe a sequence of apparent “gatekeeper” phosphorylations, which affect both hTau toxicity and neuronal dysfunction in Drosophila that could also serve as disease biomarkers in patients (Papanikolopoulou and Skoulakis, 2015).

A hypothesis put forward before (Papanikolopoulou and Skoulakis, 2015), suggested that Ser^262^ is required for Ser^238^ occupation, which was verified experimentally herein (Figure 4). Importantly, generation of the single mutants on Ser^238^ and Thr^245^ revealed an important new regulatory point not evident with the STA double mutant analyzed before. Collectively, the toxicity (Figures 2, 3) and learning (Figure 5) data in the context of the phospho-profile analyses (Figure 4), strongly indicate that Thr^245^ phosphorylation attenuates or blocks Ser^262^ occupation, which in turn is required for Ser^238^ phosphorylation leading to toxicity. Therefore, Ser^262^ phosphorylation precedes and acts as a “gatekeeper” to Ser^238^ occupation, which promotes hTau-dependent premature mortality or decreased resistance to oxidative stress, two very likely distinct manifestations of hTau toxicity.

Currently we do not know the mechanism or trigger of Thr^245^ dephosphorylation, as well as the speed and mechanism of the consequent Ser^262^ occupation, although it is likely mediated by conformational changes. As the resultant learning deficits require time after hTau expression to be manifested (Papanikolopoulou et al., 2010; Papanikolopoulou and Skoulakis, 2015), the process is likely relatively slow. Since Ser^262^ occupation precedes that of Ser^238^, this may account for the reported significant delay in phosphorylation of the latter (Papanikolopoulou and Skoulakis, 2015), a potential link to the age dependent manifestation of degenerative Tauopathies in humans as well. This is turn suggests that monitoring Ser^238^ occupation may be a useful biomarker of disease progression.

Domains such as the highly conserved microtubule binding repeats and the amino terminal region of the protein appear functionally specialized (Trushina et al., 2019). Notably, the work described herein suggests IDR/conformation-dependent interactions with the 24 aminoacids that separate Ser^238^ just amino-terminal to the first microtubule binding repeat to Ser^262^ within it, with significant roles in hTau-mediated toxicity and dysfunction. Therefore, we propose that this region defines a new potential hTau domain of importance to toxicity and neuronal dysfunction. This may be reflected in phospho-profiles of Tau fragments in Cerebrospinal fluid (CSF) samples from Alzheimer’s disease (AD) patients that show significant enrichment in Ser^238^ and Ser^262^ occupation (Russell et al., 2017). Moreover, an independent study indicated that phosphorylated Ser^238^ and Ser^262^ appear specifically associated with pathological Tau in AD patients (Martin et al., 2013). We propose that this “toxicity domain” contributes to Tau structural plasticity, as the occupation state of Ser^238^, Thr^245^, and Ser^262^ affects the phosphorylation state of additional proximal sites such as Ser^202^/Thr^205^ (AT8), Thr^212^/Ser^214^ (AT100), but also the distant Ser^396^ at the far carboxy-terminus of the protein (Figure 4), which when phosphorylated is also enriched in the CSF from AD patients (Russell et al., 2017). As all the sites under consideration are in invariant hTau regions, the regulatory mechanisms we propose are pertinent to all isoforms.

**FIGURE 6:**
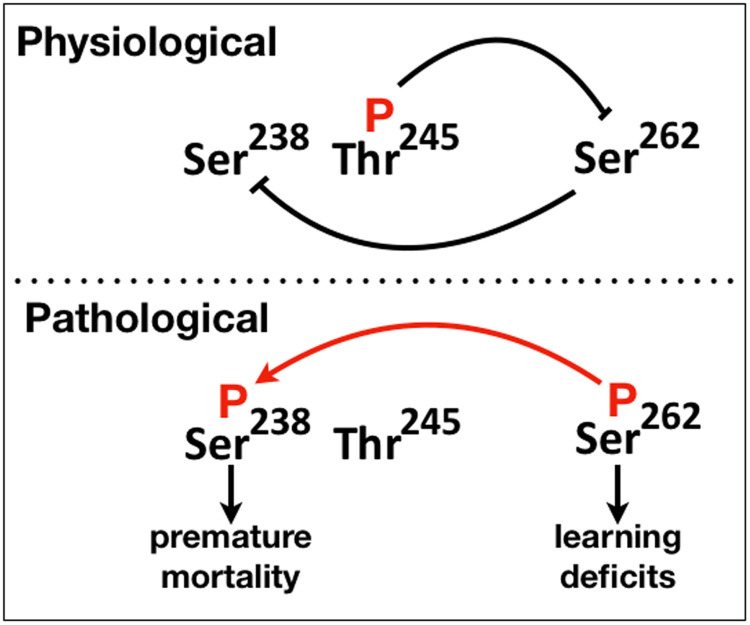
A schematic of the proposed phosphorylation-mediated interaction between “suppressive” and “facilitatory gatekeeper” phosphosite interactions proximal to and within the first microtubule binding repeat of hTau. Blunt lines represent “suppressive” while the red arrow “facilitatory” interactions. The direction of the arrow indicates that Ser^238^ is phosphorylated after occupation of Ser^262^.

Importantly, Thr^245^ phosphorylation appears to be inhibitory not only to Ser^262^ and consequently to Ser^238^ occupation, but also on Ser^202^/Thr^205^ and Thr^212^/Ser^214^ (Figure 4B) and perhaps it represents a broader suppressor of toxicity and neuronal dysfunction (Figure 6). Hence, occupation of this site appears to act as a “gatekeeper” against additional phosphorylations linked to pathologies. In agreement with this notion, phosphorylated Thr^245^ appears exclusively in physiological human brain lysates (Martin et al., 2013). To our knowledge, the only other phosphorylation reported to decrease Tau/Aβ-induced toxicity is on Thr^205^ (Ittner et al., 2016). This contrasts with the apparent role of Ser^262^ phosphorylation as an enabler of phosphorylation at many additional sites, probably a lot more than we have tested herein (Figure 4C), including the essential for toxicity Ser^238^. To that end ongoing efforts aim at generating an anti p-Thr^245^ antibody, which along with either pSer^238^and pSer^262^ may be useful biomarkers in monitoring progression of Tauopathies.

Collectively therefore, there appear to be two types of “gatekeeper” phosphosites, those that when occupied suppress additional phosphorylations such as pThr^245^ and others like pSer^262^ that enable them. This is reflected in the schematic on Figure 6, where we propose that abrogation or attenuation of Thr^245^ phosphorylation removes the effect of the toxicity and dysfunction “suppressing gatekeeper,” enabling occupation of the “facilitatory gatekeeper” phospho-Ser^262^ (Nishimura et al., 2004; Papanikolopoulou and Skoulakis, 2015). In agreement with others (Trushina et al., 2019), we posit that these changes in hTau toxicity are manifestations of the IDR-mediated structural plasticity of the protein with local or broad conformational changes favored by the presence or absence of particular phosphorylations leading to physiological or pathogenic states.

One notable result from the work herein is that the single charge contributed by Glu replacing the relevant Serines and Threonines does not mimic the double charge of the phosphate group in the case of Ser^238^ (Figures 2B, 3, 4A, 5C) and Ser^262^ (Figures 4C, 5E). This indicates that the proposed local structural plasticity is at least in part mediated and possibly stabilized by weak charge based interactions, a proposed characteristic of intrinsically disordered proteins (Trushina et al., 2019). Interestingly, our data suggests that with respect to these sites, Glu substitution may in fact mimic blocking phosphorylation at these sites (Figures 2B, 3, 4A,C, 5C,E), possibly by occlusion and this approach should be used with caution.

Although they are obvious pharmaceutical targets, it remains a challenge to identify and interfere with the phosphatases and kinases that target specific phosphoepitopes in part because of overlapping consensus sequences and participation in many other physiological cellular processes (Martin et al., 2013). For Thr^245^, whose phosphorylation appears to function as “suppressive gatekeeper” to subsequent pathology-linked phosphorylations, the therapeutic challenge is to maintain its occupation. In addition, development of an anti-phospho Thr^245^ antibody for early detection of its dephosphorylated state may be a good disease prognostic biomarker.

## Data Availability Statement

The raw data supporting the conclusions of this article will be made available by the authors, without undue reservation.

## Author Contributions

IK and EV performed research and prepared the manuscript. EV wrote the manuscript initial draft. KP performed research and guidance. ES directed the research and wrote the manuscript. All authors contributed to the article and approved the submitted version.

## Conflict of Interest

The authors declare that the research was conducted in the absence of any commercial or financial relationships that could be construed as a potential conflict of interest.
