# Supplementary material for: Functional Interactions of Tau Phosphorylation Sites That Mediate Toxicity and Deficient Learning in Drosophila melanogaster
Source: Front Mol Neurosci. 2020 Oct 21;13:569520. doi: 10.3389/fnmol.2020.569520 (PMC7609872; doi:10.3389/fnmol.2020.569520)
Supplement: Supplementary file 1 [file Image_1.pdf]

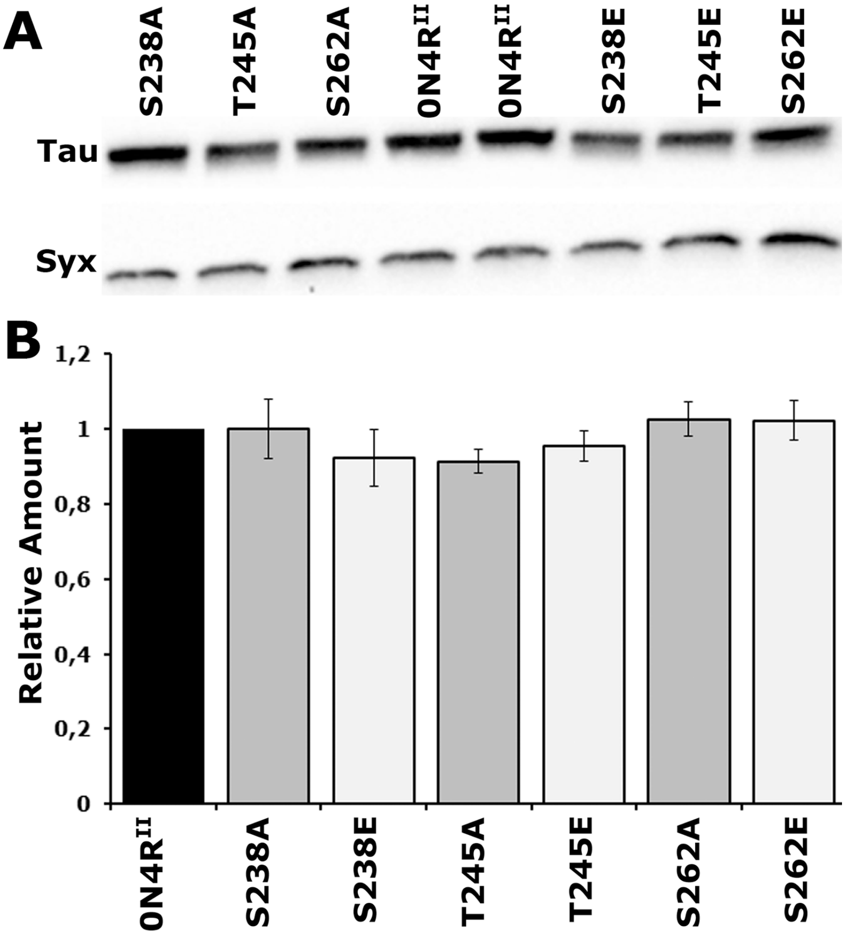


C

| Genotype | Mean ± SEM | p |
|---|---|---|
| ANOVA $F_{(6,139)}$ = 0.7679, p=0.5969 | | |
| Elav;Ras2>0N4R$^{II}$ | 1 | |
| Elav;Ras2>S238A | 0.999 ± 0.078 | 1 |
| Elav;Ras2>S238E | 0.922 ± 0.075 | 0.925 |
| Elav;Ras2>T245A | 0.914 ± 0.032 | 0.807 |
| Elav;Ras2>T245E | 0.956 ± 0.040 | 0.993 |
| Elav;Ras2>S262A | 1.026 ± 0.046 | 0.999 |
| Elav;Ras2>S262E | 1.023 ± 0.052 | 0.999 |
| | | |

**Supplemental Figure 1. Mutant and control transgenes inserted at 55B2 are expressed equivalently.**
**A)** Representative Western blot demonstrating the levels of 0N4R$^{II}$ and single Tau mutant accumulation under Elav;Ras probed with the T46 anti-Tau antibody.
**B)** For the quantifications below, levels of the protein were normalized using the Syntaxin (Syx) loading control and shown as a ratio of their means ±S.E.M. relative to their respective levels in fly heads expressing 0N4R$^{II}$, which is set to 1. Statistical details presented in C.
