# Supplementary material for: Functional Interactions of Tau Phosphorylation Sites That Mediate Toxicity and Deficient Learning in Drosophila melanogaster
Source: Front Mol Neurosci. 2020 Oct 21;13:569520. doi: 10.3389/fnmol.2020.569520 (PMC7609872; doi:10.3389/fnmol.2020.569520)
Supplement: Supplementary file 2 [file Image_2.pdf]

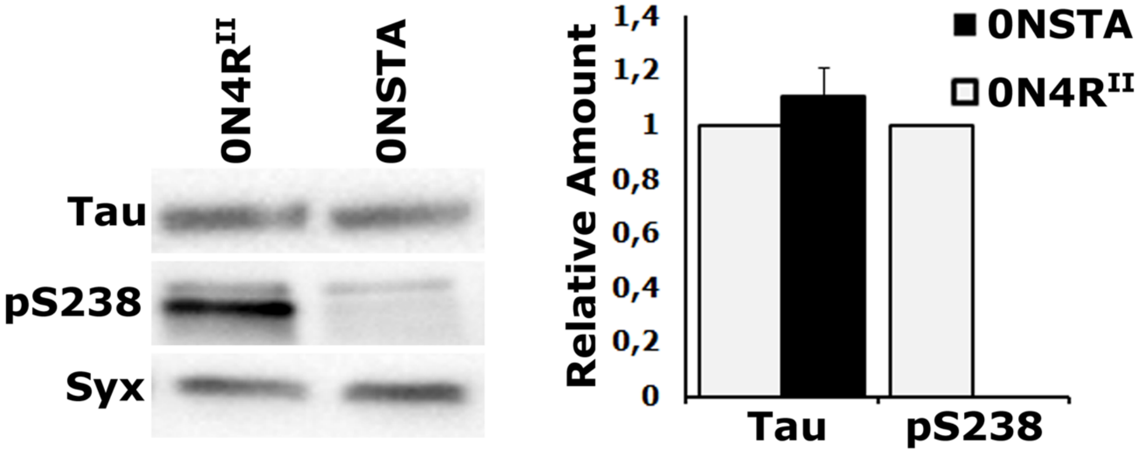

Supplemental Figure 2. 0N4R^{STA} is expressed at the same level as 0N4R^{II} and lacks the pSer^{238} epitope.

Representative Western blots from head lysates of flies accumulating 0N4R^{II} and 0NSTA probed with the antibodies indicated on the left. The level of syntaxin (Syx) in the lysates was used as control for quantifications. Quantification of at least three independent blots and extracts are shown on the right. The syntaxin-normalized level of 0N4R^{II} for each quantification was fixed to 1. The bars represent the mean ± SEM relative levels of 0NSTA over that of the 0N4R^{II} control. Dunnett's tests indicated significant difference from control for the phosphosite S238 ($p<0.0001$) and no difference in accumulation of Tau ($p=0.4945$).
