# Supplementary material for: Functional Interactions of Tau Phosphorylation Sites That Mediate Toxicity and Deficient Learning in Drosophila melanogaster
Source: Front Mol Neurosci. 2020 Oct 21;13:569520. doi: 10.3389/fnmol.2020.569520 (PMC7609872; doi:10.3389/fnmol.2020.569520)
Supplement: Supplementary file 3 [file Table_1.pdf]

Supplemental Table 1.

	Genotype	Mean ± SEM
Fig 1B	Elav>0N4R^{II}	0,396 ± 0,073
	Ras2>0N4R^{II}	0,264 ± 0,091
	Elav;Elav>0N4R^{II}	0,656 ± 0,100
	Elav;Ras2>0N4R^{II}	1,108 ± 0,039
	**ANOVA: $F_{(4,15)}$= 15.331, p= 2.8703 x 10^{-4}**	
		Dunnetts' p
	Elav>0N4R	
	Elav>0N4R^{II}	**3.5 x 10^{-3}**
	Ras2>0N4R^{II}	**8.4 x 10^{-4}**
	Elav;Elav>0N4R^{II}	**0,081**
	Elav;Ras2>0N4R^{II}	**0,826**

	Genotype	Mean ± SEM
Fig 1C	w^{1118}>0N4R^{II}	73.745 ± 0.954
	Elav>0N4R^{II}	62.497 ± 1.349
	Elav;Elav>0N4R^{II}	58.454 ± 1.511
	Elav;Ras2>0N4R^{II}	56.301 ± 1.643
	**ANOVA: $F_{(3,47)}$= 31.273, p= 5.530 x 10^{-11}**	

**0N4R^{II} >**	**w^{1118}**		**Elav**		**Elav;Elav**		**Elav;Ras2**	
	t-Ratio	p	t-Ratio	p	t-Ratio	p	t-Ratio	p
**w^{1118}**			32.78	**8.5x10^{-7}**	60.60	**8.2x10^{-10}**	78.86	**2.2x10^{-11}**
**Elav**	32.78	**8.5x10^{-7}**			4.23	**0.045**	9.95	**0.002**
**Elav;Elav**	60.60	**8.2x10^{-10}**	4.23	**0.045**			1.20	**0.279**
**Elav;Ras2**	78.86	**2.2x10^{-11}**	9.95	**0.002**	1.20	**0.279**		

Supplemental Table 1. Statistical details from Fig 1B and C.

Fig 1B. The means and SEMs are shown of the densitometrically determined ratio of hTau normalized for loading by Syntaxin for the indicated genotypes from 5 independent experiments. Following the indicated significant ANOVA the means we compared using Dunnett's tests with the means of the accumulation of the randomly inserted 0N4R encoding transgene.

Fig 1C. The means and SEMs for learning performance of the indicated genotypes are shown (n $\geq$ 9). Following the indicated significant ANOVA the means we compared using the indicated planned multiple comparisons.
