# Supplementary material for: Functional Interactions of Tau Phosphorylation Sites That Mediate Toxicity and Deficient Learning in Drosophila melanogaster
Source: Front Mol Neurosci. 2020 Oct 21;13:569520. doi: 10.3389/fnmol.2020.569520 (PMC7609872; doi:10.3389/fnmol.2020.569520)
Supplement: Supplementary file 4 [file Table_2.pdf]

Supplemental Table 2

FIGURE 2A					
**Wilcoxon/Kruskal-Wallis**			**Means comparison (Steel with control)**		
**DAY**	**χ^2, (DF,count)**	**p > χ^2**	**Genotype**	**z**	**p**
**1**	0.000 (2,17)	1.000	Elav;Ras2>0NSTA		
			Elav;Ras2>0N4R^{II}		
**9**	4.851 (2,17)	0.0884	Elav;Ras2>0NSTA		
			Elav;Ras2>0N4R^{II}		
**13**	6.736 (2,17)	0.0344	Elav;Ras2>0NSTA	0.2991	0.9368
			Elav;Ras2>0N4R^{II}	-2.4960	0.0237
**17**	12.029 (2,17)	0.0024	Elav;Ras2>0NSTA	-0.4854	0.8434
			Elav;Ras2>0N4R^{II}	-3.5005	0.0009*
**21**	18.748 (2,17)	<0.0001	Elav;Ras2>0NSTA	-0.1071	0.9916
			Elav;Ras2>0N4R^{II}	-4.0369	0.0001*
**23**	23.222 (2,17)	<0.0001	Elav;Ras2>0NSTA	-0.4987	0.8357
			Elav;Ras2>0N4R^{II}	-4.5185	<0.0001*
**25**	23.710 (2,17)	<0.0001	Elav;Ras2>0NSTA	-1.1512	0.4072
			Elav;Ras2>0N4R^{II}	-4.5403	<0.0001*
**27**	26.808 (2,17)	<0.0001	Elav;Ras2>0NSTA	-1.3162	0.3156
			Elav;Ras2>0N4R^{II}	-4.6813	<0.0001*
**29**	21.792 (2,17)	<0.0001	Elav;Ras2>0NSTA	-0.9507	0.5346
			Elav;Ras2>0N4R^{II}	-4.6309	<0.0001*
**31**	8.003 (2,17)	0.0183	Elav;Ras2>0NSTA	1.7182	0.1518
			Elav;Ras2>0N4R^{II}	-1.2432	0.3545
**33**	9.000 (2,17)	0.0111	Elav;Ras2>0NSTA	1.4759	0.2404
			Elav;Ras2>0N4R^{II}	-1.7498	0.1424
**35**	15.806 (2,17)	0.0004	Elav;Ras2>0NSTA	2.8776	0.0077
			Elav;Ras2>0N4R^{II}	0.0000	<0.0001*
**37**	10.836 (2,17)	0.0044	Elav;Ras2>0NSTA	2.3487	0.0353
			Elav;Ras2>0N4R^{II}	0.0000	<0.0001*

FIGURE 2B

Wilcoxon/Kruskal-Wallis			Means comparison (Steel with control)		
**DAY**	χ^2, **(DF,count)**	**p >** χ^2	**Genotype**	**z**	**p**
**1**	0.000 (3,17)	1.000	Elav;Ras2>0N4R^{II}		
			Elav;Ras2>S238A		
			Elav;Ras2>S238E		
**9**	0.803 (3,17)	0.8474	Elav;Ras2>0N4R^{II}		
			Elav;Ras2>S238A		
			Elav;Ras2>S238E		
**13**	4.485 (3,17)	0.2136	Elav;Ras2>0N4R^{II}		
			Elav;Ras2>S238A		
			Elav;Ras2>S238E		
**17**	0.735 (3,17)	0.8649	Elav;Ras2>0N4R^{II}		
			Elav;Ras2>S238A		
			Elav;Ras2>S238E		
**21**	2.184 (3,17)	0.5351	Elav;Ras2>0N4R^{II}		
			Elav;Ras2>S238A		
			Elav;Ras2>S238E		
**23**	12.612 (3,17)	0.0056	Elav;Ras2>0N4R^{II}	-2.5828	0.0269
			Elav;Ras2>S238A	-3.3387	0.0024*
			Elav;Ras2>S238E	-2.2527	0.0643
**25**	17.394 (3,17)	0.0006	Elav;Ras2>0N4R^{II}	-3.3442	0.0024*
			Elav;Ras2>S238A	-3.5856	0.0010*
			Elav;Ras2>S238E	-3.0621	0.0063
**27**	13.619 (3,17)	0.0035	Elav;Ras2>0N4R^{II}	-3.1389	0.0049*
			Elav;Ras2>S238A	-2.5576	0.0289
			Elav;Ras2>S238E	-2.9983	0.0053*
**29**	19.763 (3,17)	0.0002	Elav;Ras2>0N4R^{II}	-3.3843	0.0021*
			Elav;Ras2>S238A	-3.2606	0.0032*
			Elav;Ras2>S238E	-3.9454	0.0002*
**30**	19.554 (3,17)	0.0002	Elav;Ras2>0N4R^{II}	-3.4931	0.0014*
			Elav;Ras2>S238A	-3.2680	0.0031*
			Elav;Ras2>S238E	-3.7314	0.0006*
**31**	6.010 (3,17)	0.0111	Elav;Ras2>0N4R^{II}		
			Elav;Ras2>S238A		
			Elav;Ras2>S238E		
**33**	1.526 (3,17)	0.6763	Elav;Ras2>0N4R^{II}		
			Elav;Ras2>S238A		
			Elav;Ras2>S238E		
**35**	2.778 (3,17)	0.4272	Elav;Ras2>0N4R^{II}		
			Elav;Ras2>S238A		
			Elav;Ras2>S238E		

FIGURE 2C					
**Wilcoxon/Kruskal-Wallis**			**Means comparison (Steel with control)**		
**DAY**	**χ^2, (DF,count)**	**p > χ^2**	**Genotype**	**z**	**p**
**1**	0.000 (3,17)	1.000	Elav;Ras2>0N4R^{II}		
			Elav;Ras2>T245A		
			Elav;Ras2>T245E		
**9**	1.975 (3,17)	0.5777	Elav;Ras2>0N4R^{II}		
			Elav;Ras2>T245A		
			Elav;Ras2>T245E		
**13**	5.537 (3,17)	0.1364	Elav;Ras2>0N4R^{II}		
			Elav;Ras2>T245A		
			Elav;Ras2>T245E		
**17**	4.936 (3,17)	0.1766	Elav;Ras2>0N4R^{II}		
			Elav;Ras2>T245A		
			Elav;Ras2>T245E		
**21**	13.256 (3,17)	0.0041	Elav;Ras2>0N4R^{II}	-0.8529	0.7300
			Elav;Ras2>T245A	-2.2792	0.0600
			Elav;Ras2>T245E	1.6196	0.2496
**23**	26.712 (3,17)	<0.0001	Elav;Ras2>0N4R^{II}	-2.5828	0.0269
			Elav;Ras2>T245A	-4.2948	<0.0001*
			Elav;Ras2>T245E	-0.3894	0.9628
**25**	32.021 (3,17)	<0.0001	Elav;Ras2>0N4R^{II}	-3.3443	0.0024*
			Elav;Ras2>T245A	-4.6484	<0.0001*
			Elav;Ras2>T245E	-1.6225	0.2483
**27**	26.331 (3,17)	<0.0001	Elav;Ras2>0N4R^{II}	-3.1388	0.0049*
			Elav;Ras2>T245A	-4.0639	0.0001*
			Elav;Ras2>T245E	-1.5834	0.2665
**29**	31.289 (3,17)	<0.0001	Elav;Ras2>0N4R^{II}	-3.3841	0.0021*
			Elav;Ras2>T245A	-4.7469	<0.0001*
			Elav;Ras2>T245E	-1.2106	0.4820
**30**	31.637 (3,17)	<0.0001	Elav;Ras2>0N4R^{II}	-3.4931	0.0014*
			Elav;Ras2>T245A	-4.7034	<0.0001*
			Elav;Ras2>T245E	-1.9241	0.1365
**31**	16.445 (3,17)	0.0009	Elav;Ras2>0N4R^{II}	-1.4835	0.3170
			Elav;Ras2>T245A	-2.8833	0.0111
			Elav;Ras2>T245E	1.0863	0.5671
**33**	6.322 (3,17)	0.0970	Elav;Ras2>0N4R^{II}		
			Elav;Ras2>T245A		
			Elav;Ras2>T245E		
**35**	1.975 (3,17)	0.5776	Elav;Ras2>0N4R^{II}		
			Elav;Ras2>T245A		
			Elav;Ras2>T245E		

Supplemental Table 2. Statistical details from Fig 2.

Survival results from 17 independent determinations were compared with Wilcoxon/Kruskal-Wallis tests for the indicated days. When the χ^2 was significant, significant differences from the survival of Elav;Ras2>w^{1118} controls were investigated using Steel with control tests whose z ratio and p values are shown. Significant differences from controls are highlighter in light gray and emphasized by asterisks.
