# Supplementary material for: Functional Interactions of Tau Phosphorylation Sites That Mediate Toxicity and Deficient Learning in Drosophila melanogaster
Source: Front Mol Neurosci. 2020 Oct 21;13:569520. doi: 10.3389/fnmol.2020.569520 (PMC7609872; doi:10.3389/fnmol.2020.569520)
Supplement: Supplementary file 5 [file Table_3.pdf]

Supplemental Table 3.

ANOVA: $F_{(6,104)}=19.7546$, $p=5.3261 \times 10^{-15}$		
**Tau variants vs w^{1118}**		
**Genotype**	**t-Ratio**	**p**
$0N4R^{II}$	33.6099	8.2167×10^{-8}
0NSTA	0.1562	0.6934
S238A	67.8144	7.9778×10^{-13}
S238E	23.1285	5.4824×10^{-6}
T245A	49.6900	2.5435×10^{-10}
T245E	17.7771	5.5375×10^{-5}
**Glu vs Ala mutants**		
**Genotype**	**t-Ratio**	**p**
S238A vs S238E	11.7356	8.9715×10^{-4}
T245A vs T245E	8.0248	5.60149×10^{-3}
**Tau variants vs $0N4R^{II}$**		
**Genotype**	**t-Ratio**	**p**
0NSTA	29.1829	4.6172×10^{-7}
S238A	5.9416	0.0165
S238E	0.9765	0.3254
T245A	1.5667	0.2136
T245E	2.4999	0.1170
**Tau variants vs 0NSTA**		
**Genotype**	**t-Ratio**	**p**
S238A	61.4604	5.5674×10^{-12}
S238E	19.4828	2.6125×10^{-5}
T245A	44.2736	1.6464×10^{-9}
T245E	14.6001	2.3336×10^{-4}

Supplemental Table 3. Statistical details from Fig 3

Mean mortalities after 24 hrs of exposure to 30mM methyl viologen of the indicated genotypes were compared with that of Elav;Ras2>w^{1118} control animals (n=15) following the indicated highly significant ANOVA with planned multiple comparisons as indicated.
