# Supplementary material for: Functional Interactions of Tau Phosphorylation Sites That Mediate Toxicity and Deficient Learning in Drosophila melanogaster
Source: Front Mol Neurosci. 2020 Oct 21;13:569520. doi: 10.3389/fnmol.2020.569520 (PMC7609872; doi:10.3389/fnmol.2020.569520)
Supplement: Supplementary file 6 [file Table_4.pdf]

**SUPPLEMENTAL TABLE 4**

| | Antibody | ANOVA | Dunnett's p | |
|---|---|---|---|---|
| Fig 4A | **AT8** | $F_{(2,77)}$=0.4568, p=0.6350 | **S238A** | **S238E** |
| | | | | |
| | **AT100** | $F_{(2,57)}$=58.0959, p=2.7491 x $10^{-14}$ | **S238A** | **S238E** |
| | | | 2.3578 x $10^{-4}$ | 1.4981 x$10^{-8}$ |
| | **pS262** | $F_{(2,39)}$=10.8692, p=1.9349 x $10^{-4}$ | **S238A** | **S238E** |
| | | | 4.9992 x$10^{-4}$ | 4.8214 x$10^{-4}$ |
| | **pS396** | $F_{(2,85)}$= 9.0999, p=2.6715 x $10^{-4}$ | **S238A** | **S238E** |
| | | | 1.6780 x $10^{-3}$ | 0.7914 |
| | **pS238** | | **S238A** | **S238E** |
| | | | | |
| Fig 4B | **AT8** | $F_{(2,62)}$=128.1423, p=2.1988 x$10^{-22}$ | **T245A** | **T245E** |
| | | | 1.7794 x $10^{-8}$ | 1.7794 x $10^{-8}$ |
| | **AT100** | $F_{(2,76)}$=51.8107, p=8.5164 x$10^{-15}$ | **T245A** | **T245E** |
| | | | 5.3832 x $10^{-5}$ | 1.3669 x$10^{-8}$ |
| | **pS262** | $F_{(2,54)}$=15.4009, p=5.5852 x $10^{-6}$ | **T245A** | **T245E** |
| | | | 2.6969 x $10^{-3}$ | 0.3519 |
| | **pS396** | $F_{(2,62)}$=0.6312, p=0.5354 | **T245A** | **T245E** |
| | | | | |
| | **pS238** | $F_{(2,25)}$=6.0830, p=7.2933 x$10^{-3}$ | **T245A** | **T245E** |
| | | | 0.9999 | 0.0138 |
| Fig 4C | **AT8** | $F_{(2,68)}$=8.9678, p=3.587 x$10^{-4}$ | **S262A** | **S262E** |
| | | | 2.0874 x $10^{-4}$ | 1.3759 x $10^{-3}$ |
| | **AT100** | $F_{(2,57)}$=282.5058, p=1.1721 x$10^{-29}$ | **S262A** | **S262E** |
| | | | 1.4967 x$10^{-8}$ | 1.4967 x$10^{-8}$ |
| | **pS262** | | **S262A** | **S262E** |
| | | | | |
| | **pS396** | $F_{(2,64)}$=280.1379, p= 8.9264 x $10^{-32}$ | **S262A** | **S262E** |
| | | | 1.8071 x$10^{-8}$ | 1.8071 x $10^{-8}$ |
| | **pS238** | $F_{(2,20)}$=68.8786, p= 1.96561 x$10^{-9}$ | **S262A** | **S262E** |
| | | | 4.8748 x $10^{-9}$ | 4.8230 x $10^{-9}$ |

**Supplemental Table 4. Statistical details from Fig 4**

ANOVAs and Dunnett's tests p's for the ratios of densitometrically determined level of each of the designated phosphoepitopes and total hTau normalized for loading with Syntaxin for the indicated $Ser^{238}$ and $Thr^{245}$ mutants relative to the ratio in $0N4R^{II}$. When experimental bands were zero or near zero ANOVA was not performed. When ANOVA was not significant further comparisons were not performed.
