# Supplementary material for: Functional Interactions of Tau Phosphorylation Sites That Mediate Toxicity and Deficient Learning in Drosophila melanogaster
Source: Front Mol Neurosci. 2020 Oct 21;13:569520. doi: 10.3389/fnmol.2020.569520 (PMC7609872; doi:10.3389/fnmol.2020.569520)
Supplement: Supplementary file 7 [file Table_5.pdf]

Supplemental Table 5.

Genotype	Mean ± SEM	t-Ratio	p
**Figure 5B.**	**ANOVA $F_{(4,52)}$ = 7.3803, p<0.0001**		
w^{1118}>0NSTA	78.322 ±1.593		
Elav;Ras2>0N4R^{II}	58.149 ± 3.314	24.81	$8.5x10^{-6}$
Elav;Ras2>S238A	61.783 ± 2.509	15.92	$2.2x10^{-4}$
Elav;Ras2>T245A	62.857 ± 3.076	14.58	$3.8x10^{-4}$
Elav;Ras2>0NSTA	61.820 ± 3.130	16.60	$1.7x10^{-4}$
Elav;Ras2>0N4R^{II}	58.149 ± 3.314		
Elav;Ras2>S238A	61.783 ± 2.509	0.80	0.373
Elav;Ras2>T245A	62.857 ± 3.076	1.41	0.239
Elav;Ras2>0NSTA	61.820 ± 3.130	0.86	0.357
Elav;Ras2>S238A	61.783 ± 2.509		
Elav;Ras2>T245A	62.857 ± 3.076	0.07	0.792
Elav;Ras2>0NSTA	61.820 ± 3.130	0.00	0.992
Elav;Ras2>T245A	62.857 ± 3.076		
Elav;Ras2>0NSTA	61.820 ± 3.130	0.06	0.794
**Figure 5C.**	**ANOVA $F_{(3,39)}$ = 38.5284, p<0.0001**		
w^{1118}>S238A	77.327 ± 1.141		
Elav;Ras2>w^{1118}	75.407 ± 1.949	0.56	0.456
Elav;Ras2>S238A	55.275 ± 2.028	65.63	$1.2x10^{-9}$
Elav;Ras2>S238E	60.028 ± 1.665	40.13	$2.5x10^{-7}$
Elav;Ras2>w^{1118}	75.407 ± 1.949		
Elav;Ras2>S238A	55.275 ± 2.028	74.6	$2.7x10^{-10}$
Elav;Ras2>S238E	60.028 ± 1.665	48.0	$4.1x10^{-8}$
Elav;Ras2>S238A	55.275 ± 2.028		
Elav;Ras2>S238E	60.028 ± 1.665	3.83	0.058
**Figure 5D.**	**ANOVA $F_{(3,43)}$ = 15.3415, p<0.0001**		
w^{1118}>T245A	78.126 ± 1.762		
Elav;Ras2>w^{1118}	78.049 ± 1.564	0.00	0.978
Elav;Ras2>T245A	62.098 ± 2.545	31.74	$1.5x10^{-6}$
Elav;Ras2>T245E	74.956 ± 1.734	1.14	0.29
Elav;Ras2>w^{1118}	78.049 ± 1.564		
Elav;Ras2>T245A	62.098 ± 2.545	33.72	$8.8x10^{-7}$
Elav;Ras2>T245E	74.956 ± 1.734	1.26	0.267
Elav;Ras2>T245A	62.098 ± 2.545		
Elav;Ras2>T245E	74.956 ± 1.734	21.7	$3.5x10^{-5}$

Supplemental Table 5.

**Figure 5E.**	**ANOVA $F_{(3,33)} = 2.7719$, p=0.0586**		
w^{1118}>S262A	74.279 ± 2.556		
Elav;Ras2>w^{1118}	78.280 ± 2.655	0.99	0.326
Elav;Ras2>S262A	69.640 ± 2.863	4.63	0.039
Elav;Ras2>S262E	68.073 ± 3.149	6.87	0.013
Elav;Ras2>w^{1118}	78.280 ± 2.655		
Elav;Ras2>S262A	69.640 ± 2.863	1.26	0.270
Elav;Ras2>S262E	68.073 ± 3.149	2.39	0.132
Elav;Ras2>S262A	69.640 ± 2.863		
Elav;Ras2>S262E	68.073 ± 3.149	0.15	0.698

Supplemental Table5. Statistical details from Fig 5

The means and SEMs for learning performance of the indicated genotypes are shown for n $\geq$ 9. Following the indicated significant ANOVA the means we compared using the indicated planned multiple comparisons.
